# Uric acid, an important screening tool to detect inborn errors of metabolism: a case series

**DOI:** 10.1186/s13104-017-2795-2

**Published:** 2017-09-06

**Authors:** Eresha Jasinge, Grace Angeline Malarnangai Kularatnam, Hewa Warawitage Dilanthi, Dinesha Maduri Vidanapathirana, Kandana Liyanage Subhashinie Priyadarshika Kapilani Menike Jayasena, Nambage Dona Priyani Dhammika Chandrasiri, Neluwa Liyanage Ruwan Indika, Pyara Dilani Ratnayake, Vindya Nandani Gunasekara, Lynette Dianne Fairbanks, Blanka Stiburkova

**Affiliations:** 1 Department of Chemical Pathology, Lady Ridgeway Hospital for Children, Dr Danister De Silva Mawatha, Colombo 8, Sri Lanka; 2 Neurology Unit, Lady Ridgeway Hospital for Children, Colombo, Sri Lanka; 3 Nephrology Unit, Lady Ridgeway Hospital for Children, Colombo, Sri Lanka; 4 Purine Research Laboratory, St Thomas’ Hospital, London, UK; 5 Institute of Rheumatology, Prague, Czech Republic and Institute of Inherited Metabolic Disorders, First Faculty of Medicine, Charles University, Prague, Czech Republic

**Keywords:** Uric acid, Hypouricaemic hypouricosuria, Hypouricaemic hyperuricosuria, Hyperuricaemic hyperuricosuria, Purine

## Abstract

**Background:**

Uric acid is the metabolic end product of purine metabolism in humans. Altered serum and urine uric acid level (both above and below the reference ranges) is an indispensable marker in detecting rare inborn errors of metabolism. We describe different case scenarios of 4 Sri Lankan patients related to abnormal uric acid levels in blood and urine.

**Case 1:**

A one-and-half-year-old boy was investigated for haematuria and a calculus in the bladder. Xanthine crystals were seen in microscopic examination of urine sediment. Low uric acid concentrations in serum and low urinary fractional excretion of uric acid associated with high urinary excretion of xanthine and hypoxanthine were compatible with xanthine oxidase deficiency.

**Case 2:**

An 8-month-old boy presented with intractable seizures, feeding difficulties, screaming episodes, microcephaly, facial dysmorphism and severe neuro developmental delay. Low uric acid level in serum, low fractional excretion of uric acid and radiological findings were consistent with possible molybdenum cofactor deficiency. Diagnosis was confirmed by elevated levels of xanthine, hypoxanthine and sulfocysteine levels in urine.

**Case 3:**

A 3-year-10-month-old boy presented with global developmental delay, failure to thrive, dystonia and self-destructive behaviour. High uric acid levels in serum, increased fractional excretion of uric acid and absent hypoxanthine–guanine phosphoribosyltransferase enzyme level confirmed the diagnosis of Lesch–Nyhan syndrome.

**Case 4:**

A 9-year-old boy was investigated for lower abdominal pain, gross haematuria and right renal calculus. Low uric acid level in serum and increased fractional excretion of uric acid pointed towards hereditary renal hypouricaemia which was confirmed by genetic studies.

**Conclusion:**

Abnormal uric acid level in blood and urine is a valuable tool in screening for clinical conditions related to derangement of the nucleic acid metabolic pathway.

## Background

Uric acid (UA) is the end product of degradation of purine nucleotides. Altered UA level in both serum and urine is an indispensable marker in the detection of rare inborn errors of metabolism (IEM) of purine nucleotide degradation system. This article describes different clinical scenarios of 4 Sri Lankan patients detected by analyzing UA in serum and urine in the department of chemical pathology, Lady Ridgeway Hospital for Children, Sri Lanka.

## Case presentation

### Case 1

One-and-half-year-old boy of non-related parents presented with recurrent episodes of haematuria. Ultrasound scan of the abdomen revealed a bladder calculus for which he underwent vesicolithotomy. Though physical examination showed stunted growth (weight and height were below 3rd centile) his motor and mental development was appropriate for age. He was referred to the department of chemical pathology for further investigations to find the aetiology of the stone.

Xanthine crystals were detected by microscopic examination of his urine. Biochemical analysis demonstrated low serum and urine UA. Further analysis of his urine revealed high xanthine and hypoxanthine levels (Table 1).

**Table 1:** Biochemical and molecular parameters of 4 patients

	Patient 1	Patient 2	Patient 3	Patient 4
Profile	Hypouricaemic hypouricosuria	Hypouricaemic hypouricosuria	Hyperuricaemic hyperuricosuria	Hypouricaemic hyperuricosuria
Serum uric acid (μmol/l)	50 (119–428)	45 (119–428)	525 (119–428)	97 (119–428)
Urine uric acid: creatinine (mmol/mmol)	0. 001 (0.5–1.40)	0. 01 (0.7–1.50)	2.22 (0.4–1.1)	
Fractional excretion of uric acid (%)	1.8 (15–22)	0.48 (15–22)	16 (15–22)	33 (15–22)
Xanthine: creatinine (mmol/mmol)	0.33 (<0.1)	1. 45 (<0.1)		
Hypoxanthine: creatinine (mmol/mmol)	0. 04 (<0.01)	0.104 (<0.01)		
Sulfocysteine: creatinine (μmol/mmol)		188.9 (0–10)		
Enzyme assay			Undetectable hypoxanthine–guanine phosphoribosyl transferase	
Genetic analysis			*HPRT1* gene HPRT1 c.402+1G>A (IVS5+1G>A)	*SLC22A12* gene revealed missense heterozygous transitions p.T467M
Diagnosis	Xanthinuria type I or II	Molybdenum cofactor deficiency	Lesch–Nyhan disease	Renal hypouricaemia type 1

Hypouricaemic hypouricosuria with high xanthine and hypoxanthine in urine in a child who presented with a renal stone favoured the diagnosis of xanthinuria type I or II. He was started on low purine diet and advised to increase the daily fluid consumption.

### Case 2

An 8-month-old boy born to consanguineous parents presented with intractable seizures and global developmental delay. On clinical examination he was found to have microcephaly with overriding sutures, micrognathia, large right auricle and narrow bifrontal diameter. Ophthalmological examination detected subluxation of lenses. Diffuse brain atrophy with mild encephalomalacia and prominent ventricles were evident on ultrasound scan of the brain.

Uric acid results demonstrated a hypouricaemic hypouricosuric pattern. Sulfocysteine, xanthine and hypoxanthine were elevated in urine (Table 1). The clinical and radiological suspicion of molybdenum cofactor deficiency (MOCD) was confirmed biochemically.

### Case 3

A 3-year-10-month-old boy born to consanguineous parents was noted to have episodes of excessive crying and sleep disturbances since the age of 1 month. On examination the child was found to have global developmental delay, failure to thrive (height and weight < 3rd centile) and hypertonia with prominent dystonia. Bite wounds over hands and lips with evidence of bleeding were noted, suggesting a self-destructive behaviour which had been present since the age of 2 years. Macrocytes were seen in the blood smear. MRI of the brain showed mild cerebral atrophy.

Hyperuricaemic hyperuricosuria favoured the clinical suspicion of Lesch–Nyhan disease (LND) which was confirmed by undetectable hypoxanthine–guanine phosphoribosyltransferase (HGPRT) activity (Table 1). Sequencing analysis of the gene for *HGPRT* revealed that the proband is hemizygous for the mutation HPRT1 c.402+1G>A (IVS5+1G>A).

### Case 4

A 9-year-old boy, born to non-consanguineous parents with a right side renal calculus was referred to the department of chemical pathology for further investigations. He had presented with lower abdominal pain and gross haematuria for 2 weeks duration. His physical examination had been unremarkable.

Biochemical investigations showed a hypouricaemic hyperuricosuric pattern (Table 1). The sequencing analysis of the *SLC22A12* gene revealed missense heterozygous transition p.T467M supporting the diagnosis of renal hypouricaemia type 1.

### Analytical methods

Uric acid and creatinine in blood and urine were measured in multichannel auto-analysers (KONE 30 Finland, and Beckmann Coulter AU 480 USA) in the department of chemical pathology, Lady Ridgeway Hospital, Sri Lanka.

Urinary xanthine, hypoxanthine and sulphocysteines were determined by high-performance liquid chromatography. HGPRT and adenine phosphoribosyltransferase activities in erythrocyte lysates were analysed by high-performance liquid chromatography. All these tests were performed in the Purine Research Laboratory at St Thomas’ Hospital, London, UK. High performance liquid chromatography determination of hypoxanthine and xanthine in urine were performed as described previously [1].

Mutational analysis of *HPRT1* and *SLC22A12* were carried out in the Purine Research Laboratory at St Thomas’ Hospital, London, UK and the Department of Molecular Biology and Immunogenetics, Institute of Rheumatology, Czech Republic, Prague respectively. PCR amplification of *SLC22A12* and sequence analysis was performed according to Stiburkova et al. [2].

## Discussion

**Fig. 1:**
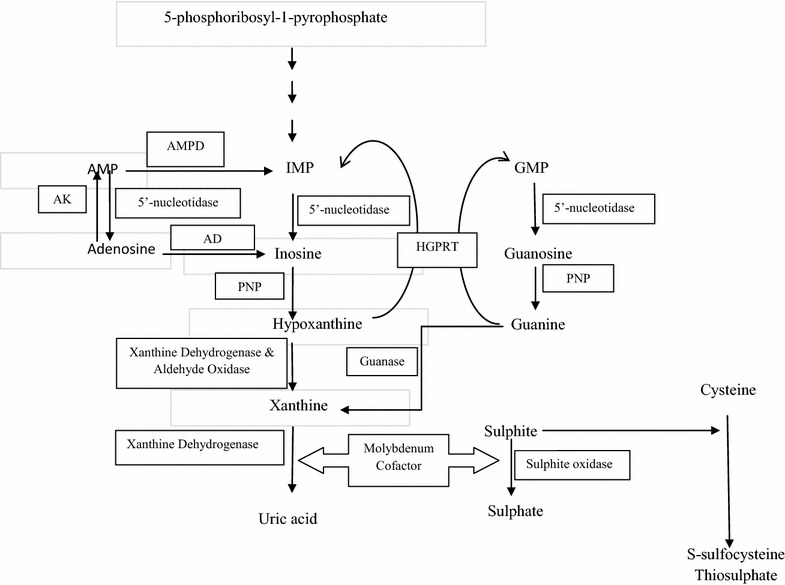
Metabolic pathway of purines. *HGPRT* hypoxanthine guanine phosphoribosyl transferase, *PNP* purine neucleosidephosphorylase, *AMP* adenosine monophosphate, *IMP* inosine monophosphate, *GMP* guanosine monophosphate, *AK* adenosine kinase, *AMPD* AMP deaminase, *AD* adenosine deaminase

Uric acid (UA) is the end product of degradation of purine nucleotides. Abnormal levels of uric acid in serum and urine may lead to detection of many defects in the purine metabolic pathway (Fig. 1) [3].

The diagnostic suspicion was not raised until low serum UA was found in two boys when they were referred to the Department of Chemical Pathology to find the aetiology for the urolithiasis. Elevated fractional excretion of uric acid (FE_UA_) led to the diagnosis of idiopathic renal hypouricaemia in 1 boy (case 4) and reduced FE_UA_ favoured xanthinuria in the other (case 1). These 2 cases highlight the importance of analyzing UA in blood and urine in patients who present with either haematuria or urinary stones. UA supported the clinical diagnosis of MOCD (case 2) and LND (case 3) in the other 2 patients who presented with neurological manifestations.

Biochemical profile of hypouricaemic hypouricosuria with elevated urinary xanthine and hypoxanthine levels were seen in 2 patients: xanthinuria type I or II in a child who presented with a bladder stone (case 1) and MOCD (case 2) in an infant who presented with neurological manifestations.

Classical xanthinuria has two forms: an isolated deficiency of xanthine oxidase/dehydrogenase (type I), or a dual deficiency (type II), in which a related molybdo enzyme, aldehyde oxidase, is deficient. Patients inheriting xanthinuria types I or II usually present with xanthine stones, xanthine crystals in the urine or acute kidney failure [4]. The two forms can be differentiated by measuring urinary oxypurinol after administering allopurinol [5]. This test was not carried out in our patient.

Molybdenum cofactor deficiency, xanthinuria type III, a rare autosomal recessive neurodegenerative disorder is characterised by a combined deficiency of molybdenum cofactor-dependent enzymes sulfite oxidase, xanthine dehydrogenase and aldehyde oxidase [6, 7]. They present with intractable seizures progressing to severe global developmental delay [8].

The hypouricaemic hypouricosuric state in both conditions is due to the deficiency of xanthine dehydrogenase enzyme involved in the catabolism of purines (Fig. 1). In MOCD, the clinical picture of sulfite oxidase deficiency, an enzyme involved in the metabolism of sulphites, dominates that of xanthine dehydrogenase [9], though elevated biochemical markers are due to deficiency of both xanthine dehydrogenase (xanthine and hypoxanthine) and sulfite oxidase (S-sulphocysteine) (Fig. 1).

In both patients, elevation of xanthine in urine was greater than that of hypoxanthine (Table 1). The preferential excretion of xanthine in urine results from extensive recycling of hypoxanthine by the enzyme HGPRT, for which xanthine is not a substrate. In addition xanthine continues to accumulate due to the conversion of guanine to xanthine by the enzyme guanase (Fig. 1) [10].

Hereditary disorders of the renal handling of urate, manifested in high urate clearance such as idiopathic renal hypouricaemia (RHUC), Fanconi and Hartnup syndromes cause hypouricaemic hyperuricosuria [11]. The diagnosis of RHUC was made in a boy who presented with episodes of haematuria and a biochemical profile of hypouricaemic hyperuricosuria (case 4). RHUC is an autosomal recessive hereditary disorder [12]. It is characterized by impaired tubular UA transport, reabsorption insufficiency and/or the acceleration of secretion [13].

The diagnosis of RHUC is based on biochemical markers: hypouricaemia and increased FEua. Confirmation of the diagnosis is accomplished by molecular analysis of the *SLC22A12* gene [14]. Currently, there are two subtypes of RHUC. Type 1 is characterized by loss of function mutations in the *SLC22A12* gene, which encodes urate transporter 1. Mutations in the *SLC22A12* gene are responsible for most cases of renal hypouricemia. In contrast, type 2 was revealed to be caused by defects in the *SLC2A9* gene, which codes transporter GLUT9 [15, 16].

Although most of the patients are asymptomatic some of them are at risk of developing UA nephrolithiasis or acute kidney injury following severe exercise [17]. On the basis of genetic analysis due to the presence of a mutation in the *SLC2A9* gene our patient (case 4) is affected by renal hypouricaemia type 1.

In our child discussed in case 3, clinical suspicion of LND arose from the presence of self-injurious behavior associated with global developmental delay and hyperuricaemic hyperuricosuric biochemical profile.

Purine pathway defects that belong to hyperuricaemic hyperuricosuria include LND, Kelley–Seegmiller syndrome, 5-phosphoribosyl-1 pyrophosphate synthetase over activity and myoadenylate deaminase deficiency [11].

LND is an X-linked recessive disorder that results from mutations in the *HPRT1*gene [18]. HGPRT, an enzyme in the purine salvage pathway, recycles the purine bases, hypoxanthine and guanine, into the usable purine nucleotide pools (Fig. 1). In the absence HGPRT, hypoxanthine and guanine cannot be recycled and, instead they are degraded to UA. The failure of purine recycling together with enhanced purine synthesis is responsible for the overproduction of UA (Fig. 1) [19].

Despite this overproduction, marked increase of the serum uric acid level is prevented by efficient renal clearance. Hence, measurement of urinary uric acid provides a more accurate estimate of total UA production [11, 18].

Our patient exhibited the typical features mentioned in the literature such as short stature, recurrent self-injury and asymptomatic macrocytosis [20].

## Conclusion

Inherited defects of purine metabolism comprise a group of disorders with very variable clinical manifestations. Altered levels of serum and urine UA levels may constitute effective markers in screening especially when expensive techniques may not be available for the detection for inborn errors of metabolic disorders of the purine pathways. Early diagnosis is beneficial to the patient and will enable targeted management and investigations.

## Abbreviations

UA: uric acid
FE_UA_: fractional excretion of uric acid
IEM: inborn errors of metabolism
MOCD: molybdenum cofactor deficiency
LND: Lesch–Nyhan disease
HGPRT: hypoxanthine–guanine phosphoribosyl transferase
RHUC: renal hypouricaemia

## References

[1] Mraz M, Hurba O, Bartl J, Dolezel Z, Marinaki A, Fairbanks L, Stiburkova B (2015). Modern diagnostic approach to hereditary xanthinuria. Urolithiasis.

[2] Stiburkova B, Sebesta I, Ichida K, Nakamura M, Hulkova H, Krylov V (2013). Novel allelic variant and evidence for a prevalent mutation in URAT1 causing renal hypouricemia: biochemical, genetics and functional analysis. Eur J Hum Genet.

[3] Van Gennip AH (1999). Defects in metabolism of purines and pyrimidines. Nederlands Tijdschrift voor Klinische Chemie.

[4] Simmonds H, Reiter S, Nishino T. Hereditary xanthinuria. Orphanet Encyclopedia. 2003. https://www.researchgate.net/publication/285222077_Hereditary_xanthinuria. Accessed July 2016.

[5] Reiter S, Simmonds HA, Zollner N, Braun SL, Knedel M (1990). Demonstration of a combined deficiency of xanthine oxidase and aldehyde oxidase in xanthinuric patients not forming oxipurinol. Clin Chim Acta.

[6] Johnson JL, Duran M, Scriver CR, Beaudet AL, Sly WS, Valle D (2001). Molybdenum cofactor deficiency and isolated sulfite oxidase deficiency. The metabolic and molecular basis of inherited disease.

[7] Vijayakumar K, Gunny R, Grünewald S, Carr L, Chong KW, DeVile C (2011). Clinical neuroimaging features and outcome in molybdenum cofactor deficiency. Pediatr Neurol.

[8] Macaya A, Brunso L, Fernandez-Castillo N, Arranz JA, Ginjaar HB, Cuenca-Leon E (2005). Molybdenum cofactor deficiency presenting as neonatal hyperekplexia: a clinical, biochemical and genetic study. Neuropediatrics.

[9] Van den Berghe G, Vincent MF, Marie S, Fernandes J, Saudubray JM, van den Berghe G, Walter JH (2006). Disorders of purine and pyrimidine metabolism. Inborn metabolic diseases.

[10] Raivio KO, Saksela M, Lapatto R. Xanthine oxidoreductase-role in human pathophysiology and in hereditary xanthinuria. In: Scriver CR, Baeudet Al, Sly WS, Valle D eds. The metabolic and molecular bases of inherited disease. 8th ed. New York: Mc-Graw Hill; 2001. p. 2639-52.

[11] Simoni RE, Gomes LNLF, Scalco FB, Oliveira CPH, Neto FRA, de Oliveira MLC (2007). Uric acid changes in urine and plasma: an effective tool in screening for purine inborn errors of metabolism and other pathological conditions. J Inherit Metab Dis.

[12] Enomoto A, Kimura H, Chairoungdua A, Shigeta Y, Jutabha P, Cha SH (2002). Molecular identification of a renal urate-anion exchanger that regulates blood urate levels. Nature.

[13] Gabrikova D, Bernasovska J, Sokolova J, Stiburkova B (2015). High frequency of SLC22A12 variants causing renal hypouricemia 1 in the Czech and Slovak Roma population; simple and rapid detection method by allele-specific polymerase chain reaction. Urolithiasis.

[14] Sebesta I, Stiburkova B, Bartl J, Ichida K, Hosoyamada M, Taylor J (2011). Diagnostic tests for primary renal hypouricemia. Nucleosides Nucleotides Nucleic Acids.

[15] Matsuo H, Chiba T, Nagamori S (2008). Mutations in glucose transporter 9 gene SLC2A9 cause renal hypouricemia. Am J Hum Genet.

[16] Dinour D, Gray NK, Campbell S, Shu X, Sawyer L, Richardson W (2010). Homozygous SLC2A9 mutations cause severe renal hypouricemia. J Am Soc Nephrol.

[17] Ohta T, Sakano T, Igarashi T, Itami N, Ogawa T, Group ARFAwRHR (2004). Exercise-induced acute renal failure associated with renal hypouricaemia: results of a questionnaire-based survey in Japan. Nephrol Dial Transpl.

[18] Jinnah HA, Friedmann T. Lesch–Nyhan disease and its variants. In: Scriver CR, Beaudet AL, Sly WS, Valle D, editors. The metabolic and molecular bases of inherited disease. 8th ed. New York: McGraw-Hill; 2001. p. 2537–60.

[19] Torres RJ, Puig JG, Jinnah HA (2012). Update on the phenotypic spectrum of Lesch–Nyhan disease and its attenuated variants. Curr Rheumatol Rep.

[20] Jinnah HA (2009). Lesch–Nyhan disease: from mechanism to model and back again. Dis Model Mech.

